# Targeting CD38 alleviates tumor-induced immunosuppression

**DOI:** 10.18632/oncotarget.22992

**Published:** 2017-12-26

**Authors:** Yu-Tzu Tai, Kenneth C. Anderson

**Affiliations:** ^1^ The Jerome Lipper Multiple Myeloma Center and LeBow Institute for Myeloma Therapeutics, Dana-Farber Cancer Institute, Harvard Medical School, Boston, MA, USA

**Keywords:** Multiple myeloma, CD38, isatuximab, daratumumab, immunomodulatory activity, bone marrow microenvironment

Targeting CD38, a common blood cell membrane receptor highly expressed on many B- and T-cell cancers, has achieved significant clinical activity with an acceptable safety profile in multiple myeloma (MM). Specifically, daratumumab (dara) was the first naked CD38 monoclonal antibody (mAb) approved for the treatment of relapsed and refractory MM (RRMM) in 2015. Importantly, it induces high response rates in two phase III trials in RRMM when combined with bortezomib or lenalidomide plus dexamethasone [1]. Due to its efficacy and lack of toxicity, naked CD38 monoclonal antibody has great promise, either as a single agent or in combination, in other hematological malignancies as well.

Isatuximab (Isa), a novel humanized IgG1-kappa CD38 mAb targeting a completely different epitope in CD38 molecule than dara, achieves significant responses when combined with lenalidomide and dexamethasone in heavily pretreated patients with RRMM [2]. Isa, like dara, induces MM cell lysis via multiple effector cell-dependent mechanisms including antibody-dependent cellular cytotoxicity (ADCC), complement dependent cytotoxicity (CDC), and antibody-dependent phagocytosis (ADCP). In contrast to dara, isa directly kills MM cells via lysosome-mediated cell death and apoptosis in the absence of Fc cross-linking agents or effector cells [3]. Furthermore, this direct toxicity is preferentially seen in myeloma cells expressing elevated levels of CD38 regardless of p53 mutations, which are common in the setting of RRMM.

Since CD38 is widely expressed on hematopoietic cells, it is crucial to study how isa impacts on various CD38-expressing subsets to influence clinical responses. We recently defined the effects of isa on myeloma-supporting osteoclasts (OCs) and immune cell subsets in the bone marrow microenvironment [4, 5]. Besides directly promoting MM cell growth and survival in the bone marrow (BM) microenvironment, OCs protect MM cells against T-cell-mediated cytotoxicity via direct inhibition of proliferating CD4+ and CD8+ T cells [4]. We first found that CD38 is upregulated during osteoclastogenesis, and that isa has limited direct cytotoxicity against OCs. Importantly, isa alleviates suppression of T cell function by OCs, along with downregulation of the immune checkpoint molecule herpesvirus entry mediator (HVEM) and the T-cell metabolism regulator indoleamine 2, 3-dioxygenase (IDO). Therefore, isa may enhance immunotherapeutic activity and mitigate bone disease by restoring T-cell function.

**Figure 1:**
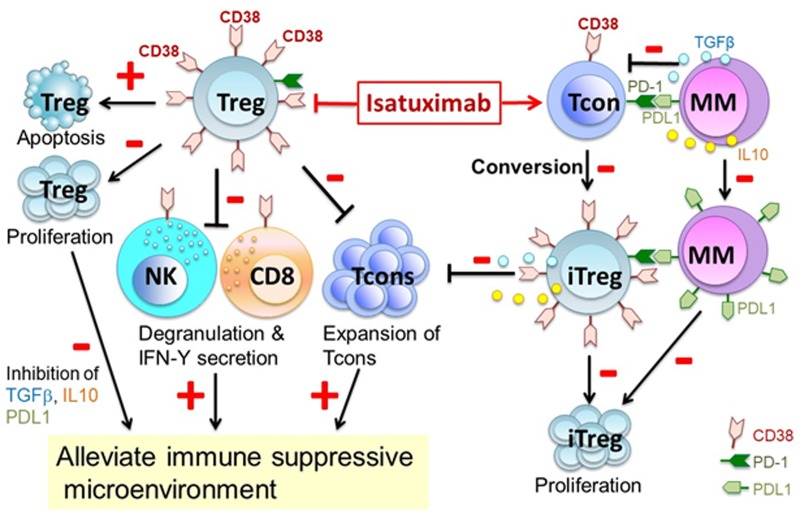
Isatuximab targets Treg to mitigate immune suppressive tumor microenvironment CD38 regulates Treg cells via interaction with MM cells and Tcons to induce an immunosuppressive tumor microenvironment. CD38 monoclonal antibody isatuximab (isa) induces apoptosis and blocks proliferation of CD38^high^ Tregs, thereby alleviating inhibition of NK and CD8+ T effector cells via up-regulating degranulation and IFNγ secretion. Thus, isa diminishes Tregs (CD38^high^) and activates effector cell (CD38^low^) function. Moreover, isa attenuates multiple myeloma (MM) cell-induced Treg (iTreg) generation from Tcons, which depends on PD1/PDL1 binding and immunosuppressive factors including TGFβ and IL10. Isa may therefore relieve immunosuppression and restore effective anti-MM immunity. Data adapted.[5]

Next, we identified significantly increased CD38 levels on the cell membrane of regulatory T cells (Tregs) (CD4+CD25^high^Foxp3+) when compared with conventional T effector cells (Tcons, CD4+CD25-) [5]. Elevated CD38 expression and CD38^high^ subsets are defined in Tregs versus Tcons, associated with significantly enhanced CD38 targeting by isa on Treg vs Tcon. As seen in CD38^high^ MM cells, isa preferentially targets Tregs vs Tcon via triggering apoptosis and decreasing proliferation (Figure 1). Furthermore, low dose (1μM) lenalidomide and pomalidomide significantly increase CD38 level on viable Tregs and percentages of CD38^high^ Tregs in culture. These results suggest that IMiDs can enhance the sensitivity of viable Tregs to Isa, resulting in enhanced NK- and CD8+ T effector cell-mediated anti-tumor immune responses.

Importantly, CD38^high^ subsets are increased on Tregs of MM patients vs normal donors. We found that MM cells can convert Tcon into Tregs in ex vivo co-cultures. These iTregs can be induced by cell-to-cell contact-dependent and -independent interactions between myeloma cells and Tcons, mimicking increased Tregs in MM patients vs normal donors. These iTregs show significantly elevated CD38 and Foxp3 levels when compared with Tcons. They still significantly decrease proliferation of Tcons, which is overcome by isa. Levels of CD38 correlate with Foxp3 in Tregs of MM patients, which inhibit proliferation of autologous Tcons. As recently reported for dara [6], isa blocks Tregs to a much greater extent than Tcons. As CD38^high^ Tregs exhibit even stronger immunosuppressive ability, targeting CD38 can abrogate this subset more effectively than CD38^low^ or negative subsets, thereby relieving the immunosuppressive microenvironment. In addition, isa decreases Foxp3 and IL10 in viable Tregs, further targeting the immunosuppressive function of Tregs. In the context of the underlying immune deficiency of MM patients, targeting Tregs by CD38 mAb to restore effective antitumor response represents a promising treatment strategy.

In addition to Treg, we found that B regulatory cells (Bregs) also express significantly higher CD38 when compared with normal T, B, NK, and monocytes [7]. Importantly, these CD19+CD24^high^CD38^high^ Bregs with immunosuppressive properties (i.e., secretion of IL-10) are defined within bone marrow (BM) more distinctly than peripheral blood (PB) in MM patients [7]. MM Bregs further abrogate NK cell-mediated ADCC against MM cells by elotuzumab. Thus, MM BM Bregs confer an immunosuppressive BM microenvironment, which may in turn impact therapeutic response and disease outcome. It is likely that isa can effectively targets these immune inhibitory CD38^high^ subsets, which were rapidly depleted by dara in a recent correlative study [6].

It remains to be determined whether differential effects of isa on Tregs vs Tcons can improve its therapeutic window. Nevertheless, early results from ongoing phase III dara-based combination trials in newly diagnosed MM patients whose immune function are relatively more intact than RRMM demonstrate significantly improved overall response rate and progression-free survival [8]. Such unexpected immune stimulatory activity of CD38 mAb may continue to transform the treatment landscape in MM and other cancers.
